# Molecular probes targeting HER2 PET/CT and their application in advanced breast cancer

**DOI:** 10.1007/s00432-023-05519-y

**Published:** 2024-03-11

**Authors:** Fang Gao, Fengxu Liu, Jun Wang, Junfang Bi, Luoping Zhai, Dong Li

**Affiliations:** 1 General Surgery Department, Third Hospital of Shanxi Medical University, Shanxi Bethune Hospital, Shanxi Academy of Medical Sciences, Tongji Shanxi Hospital, Taiyuan, Shanxi China; 2 Department of Anesthesia, Armed Police Corps Hospital in Shanxi Province, Xiaodian District, Taiyuan, Shanxi People’s Republic of China; 3 Department of Combined Traditional Chinese Medicine and West Medicine, Traditional Chinese Medicine Hospital of Shijiazhuang City, 233 Zhongshan West Road, Qiaoxi District, Shijiazhuang, Hebei China; 4 Present Address: Shanxi Province Cancer Hospital/ Shanxi Hospital Affiliated to Cancer Hospital, Chinese Academy of Medical Sciences/Cancer Hospital Affiliated to Shanxi Medical University, Taiyuan, Shanxi China; 5 Department of Nuclear Medicine, Third Hospital of Shanxi Medical University, Shanxi Bethune Hospital, Shanxi Academy of Medical Sciences, Tongji Shanxi Hospital, Taiyuan, Shanxi China

**Keywords:** HER2, PET/CT, Advanced breast cancer, Molecular probes, Clinical applications

## Abstract

**Purpose:**

Human epidermal growth factor receptor 2 (HER2)-positive breast cancer cases are among the most aggressive breast tumor subtypes. Accurately assessing HER2 expression status is vital to determining whether patients will benefit from targeted anti-HER2 treatment. HER2-targeted positron emission tomography (PET/CT) is noninvasive, enabling the real-time evaluation of breast cancer patient HER2 status with accuracy.

**Methods:**

We summarize the research progress of PET/CT targeting HER2 in breast cancer, focusing on PET/CT molecular probes targeting HER2 and their clinical application in the management of advanced breast cancer.

**Results:**

At present, a variety of different HER2 targeted molecular probes for PET/CT imaging have been developed, including nucleolin-labeled antibodies, antibody fragments, nanobodies, and peptides of various affinities, among others. HER2-targeted PET/CT can relatively accurately evaluate HER2 expression status in advanced breast cancer patients. It has good performance in the early detection of small HER2-positive lesions, evaluation of HER2 status in lesions that cannot be readily biopsied, evaluation of the heterogeneity of multiple metastases, identification of lesions with altered HER2 status, and evaluation of the efficacy of anti-HER2 drugs.

**Conclusion:**

HER2-targeted PET/CT offers a promising noninvasive approach for real-time assessment of HER2 status，which can be guide targeted treatment for HER2-positive breast cancer patients. Future prospective clinical studies will be invaluable for fully evaluating the importance of HER2-targeted molecular imaging in the management of breast cancer.

## Significance of HER2-targeted PET/CT in breast cancer

Breast cancer is among the most prevalent malignancies in the world and the fifth most common cause of cancer-associated mortality among women, with roughly 2.3 million diagnoses and 685,000 deaths throughout the world in 2020 alone (Sung et al. 2021). Mortality rates associated with recurrent metastatic breast cancer are particularly high, and advanced breast cancer patients exhibit a 5-year survival rate of just 20% (Gonzalez-Angulo et al. 2007). The administration of appropriately targeted drugs selected based on the molecular characteristics of metastatic tumors can effectively prolong the survival of these patients, underscoring the need to establish approaches to reliably determine the characteristics of target tumors.

Optimal antitumor drugs are those exhibiting a high degree of anticancer activity while causing minimal normal tissue toxicity. One of the most effective approaches to achieving such efficacy is by targeting particular proteins on the surface of actively proliferating malignant cells. In clinical practice, the most common targets of drugs aimed at the treatment of breast cancer include estrogen/progesterone receptor (ER/PR) and human epidermal growth factor receptor 2. Primary HER2-targeted treatments include monoclonal antibodies (mAbs), such as trastuzumab and pertuzumab, small molecule tyrosine kinase inhibitors including lapatinib and pyrotinib, and antibody–drug conjugates including TDM-1 and DS-8201. Anti-HER2-targeted treatment has been repeatedly linked to improvements in disease-free and overall survival in advanced breast cancer patients with HER2-positive disease. These targeted therapies are, however, limited by their higher cost and potential for side effects that can include cardiotoxicity. It is thus vital that the HER2 status of tumors be reliably determined in order to identify patients who are likely to benefit from targeted interventions (Koleva-Kolarova et al. 2017).

At present, HER2 status is primarily evaluated through the evaluation of pathological sections via immunohistochemistry (IHC) and fluorescence in situ hybridization (FISH). HER2 positivity is diagnosed when IHC (+ + +) or (+ +) and FISH assays show HER2 gene amplification. The ability to obtain a sufficient pathological specimen is thus a prerequisite for an accurate diagnosis. In many cases, however, adequate pathological samples cannot be collected as the target mass is too deep, too small, or located in proximity to large vasculature and nerves. In these patients, targeted treatment recommendations can only be made based on the HER2 status of the primary tumor. This is a problem given the potential for inconsistencies in the receptor status of primary and metastatic tumors, as evidenced by the fact that roughly 20% of metastases in patients with HER2-negative primary tumors are HER2-positive (Priedigkeit et al. 2017). Approximately 16.6% of patients with HER2-positive primary tumors may exhibit HER2-negative metastases (Sari et al. 2011). Altered receptor expression can contribute to improper or off-target treatment. In patients with multiple metastatic lesions, substantial heterogeneity can exist among these metastases whereas treatment-related decisions are generally made based on biopsy results from a single lesion, contributing to the potential for off-target treatment decisions.

PET/CT imaging is an advanced, repeatable, noninvasive approach that can enable the simultaneous systemic detection of multiple target lesions. HER2-targeted PET/CT scans provide an opportunity to specifically examine HER2 expression in multiple lesions in breast cancer patients in real time such that therapeutic planning can be performed in an individualized manner. This approach can serve as both a supplemental means of evaluating patients for whom traditional pathological analyses were conducted, as well as a primary approach to providing treatment recommendations for patients without any accessible metastatic specimens. There has been a growing interest in recent years focused on HER2-targeted PET/CT imaging in the context of the clinical management of breast cancer.

## PET/CT molecular probes targeting HER2

**Fig. 1:**
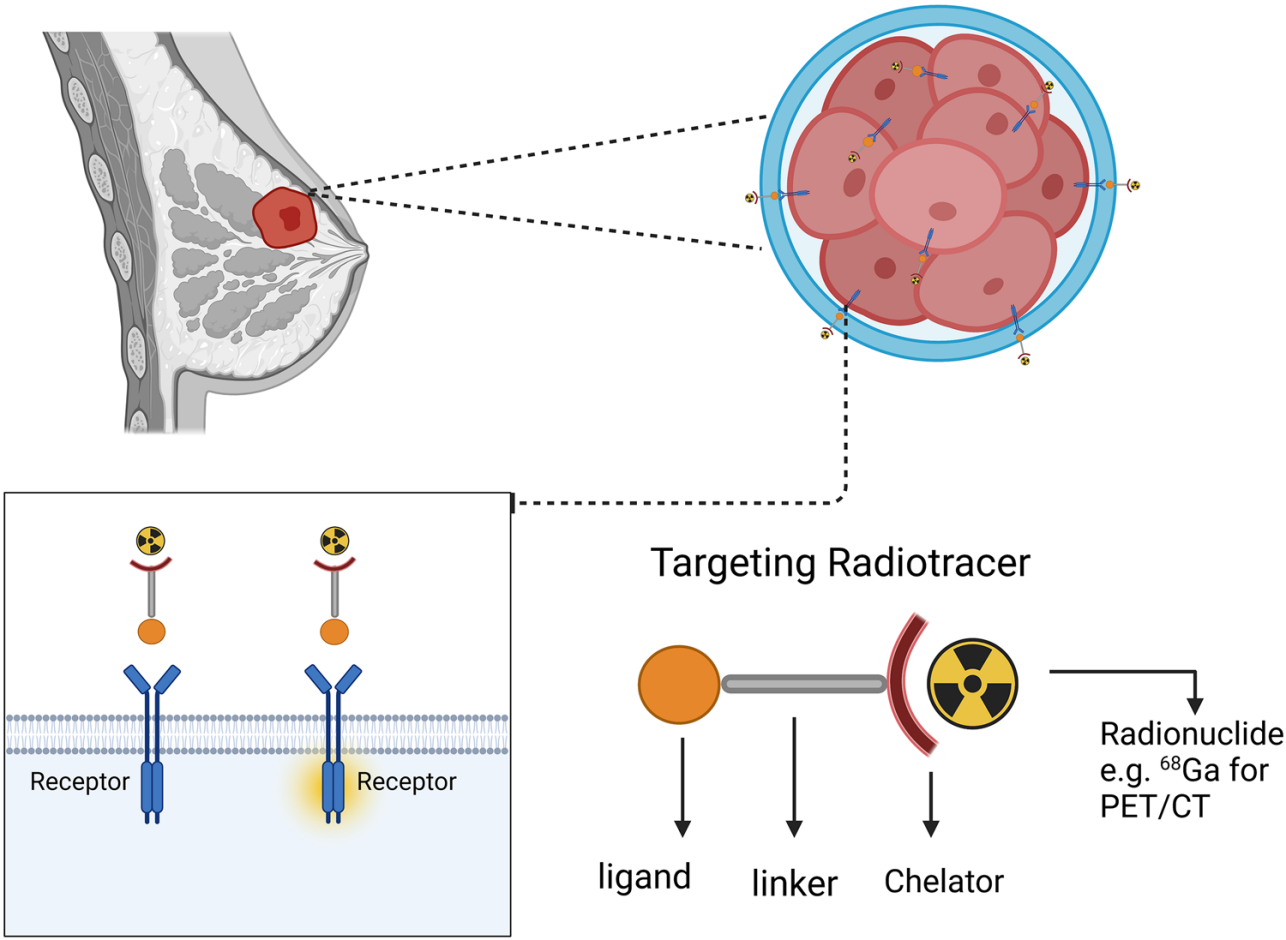
Schematic overview of targeted receptor imaging. Ligands capable of binding to specific targets that are overexpressed by breast cancer cells can be coupled via a linker domain to a chelator labeled with ^68^Ga or other radionuclides, providing an effective probe for PET/CT imaging

Several different HER2-targeting molecular probes for PET/CT imaging have been developed to date, including nucleolin-labeled antibodies, antibody fragments, nanobodies, and peptides of various affinities, among others (see Fig. 1).

### Monoclonal antibody-based molecular probes

To date, both trastuzumab and pertuzumab are monoclonal antibodies that have been subjected to radionuclide labeling. Trastuzumab recognizes a HER2 epitope located on extracellular region IV of this protein and can suppress tumor cell growth through several mechanisms including the overall downregulation of HER2 expression, the disruption of the cleavage of this HER2 extracellular region. In addition, this therapeutic mAb can inhibit the heterodimerization of HER2 and HER3, thereby disrupting intracellular PI3K signaling. Trastuzumab can also suppress angiogenesis, and trastuzumab-coated HER2-positive tumor cells can undergo antibody-dependent cellular cytotoxicity-mediated lysis upon immune cell recognition (Nahta 2012). Pertuzumab binds to a HER2 epitope located in extracellular region II of this protein and functions by inhibiting the ability of HER2 to dimerize with other growth factor receptors, exhibiting a particularly potent suppressive effect on HER2–HER3 heterodimerization (Scheuer et al. 2009).

Whole antibodies, which exhibit a molecular weight of approximately 150 kDa, often only achieve a sufficient tumor-to-blood ratio after multiple days such that they exhibit relatively limited tumor penetration and gradual clearance. As a result, short-lived radionuclides are poorly suited to antibody-mediated PET imaging. However, the relatively long half-lives of ^89^Zr and ^64^Cu make them better suited for antibody labeling and use in PET/CT imaging.

#### ^*89*^*Zr-Trastuzumab*

Owing to its prolonged 78.4 h half-life (Dijkers et al. 2010), relatively low positron energy (average: 0.396 MeV), and short positron-free travel distance, ^89^Zr can provide a high degree of spatial resolution when used for PET/CT imaging (Laforest et al. 2016). In an effort to thoroughly evaluate the most optimal dosing and timing of ^89^Zr-trastuzumab administration, Dijkers et al. (2010) evaluated 14 HER2-positive patients with metastatic breast cancer via ^89^Zr-trastuzumab PET/CT imaging. In patients that had not undergone prior trastuzumab treatment, they found that the pre-injection of a 50 mg trastuzumab dose was necessary to achieve effective imaging, whereas only a 10 mg dose was necessary for patients with a history of prior trastuzumab treatment. Ulaner et al. (2016, 2017) experimentally determined that ^89^Zr-trastuzumab administration was associated with false-positive metastatic foci, however, highlighting an important limitation of this molecular probe.

#### ^*89*^*Zr-Pertuzumab*

Ulaner et al. (2018) employed ^89^Zr-pertuzumab to detect HER2-positive metastatic breast cancer. In addition to evaluating the dosing, pharmacokinetics, and biodistribution of this labeled antibody, the authors demonstrated that this drug was not associated with any apparent toxicity. The highest dose of radiation was delivered to the liver, kidneys, and heart wall. They also found that the probe was able to facilitate tumor imaging at an optimal imaging timepoint of 5–8 days post-administration, allowing for the detection of pathologically confirmed brain metastases not detectable through other approaches, thus highlighting the value of ^89^Zr-pertuzumab.

Studies employing both trastuzumab and pertuzumab can achieve superior antitumor activity owing to the enhanced affinity of these antibodies and their complementary effects, respectively inhibiting HER2 dimerization and p95HER2 formation (Scheuer et al. 2009). In mice bearing breast tumor xenografts, ^89^Zr-pertuzumab uptake was enhanced in the presence of trastuzumab (Marquez et al. 2014). Efforts to detect HER2 status using ^89^Zr-pertuzumab may be more effective in patients undergoing trastuzumab or T-DM1 (trastuzumab emtansine) treatment.

#### ^*64*^*Cu-Trastuzumab*

^64^Cu offers a short 12.8 h half-life such that it exposes patients to a lower dose of radiation as compared to ^89^Zr-trastuzumab (Mortimer et al. 2014). Mortimer et al. (2014) demonstrated the ability of ^64^Cu-DOTA-trastuzumab to detect HER2-positive lesions. Trastuzumab pretreatment (45 mg) was sufficient to reduce hepatic uptake by ~ 75% without any impact on tumoral uptake. In vivo*,* this probe yielded a radiation uptake dose similar to that of ^18^F-FDG. While these results are promising, much as with ^89^Zr-trastuzumab, ^64^Cu-DOTA-trastuzumab can be taken up by HER2-negative tumors, and owing to the 46 h half-life of ^64^Cu-DOTA-trastuzumab in the blood such that imaging must be performed within 48 h post-administration, contributing to suboptimal imaging contrast (Carrasquillo et al. 2019).

### Fab and F(ab)_2_ antibody fragment-based probes

As mAbs exhibit a high molecular weight, they are cleared relatively slowly from the blood such that visualization is best performed 4–8 days following administration (Laforest et al. 2016). These antibodies can also be nonspecifically taken up by tumors as a result of the enhanced permeability and retention effect (EPR), limiting their clinical utility (Mendler et al. 2015). These mAbs also expose health organs to high levels of radiation and can reportedly yield false-positive findings in certain patients (Ulaner et al. 2017). To overcome this issue, proteases can be used to digest whole mAbs to isolate the Fab domains. The papain-mediated digestion of an IgG molecule yields a single Fc domain fragment and two Fab domain fragments, whereas pepsin-mediated IgG digestion yields two Fab domains linked by a hinge domain [F(ab)_2_] and one Fc domain fragment. Owing to their lower molecular weight, Fab and F(ab)_2_ fragments can be rapidly cleared at earlier time points such that superior tumor contrast can be achieved, while also eliminating the Fc domain such that these imaging probes have no impact on anti-Fc-mediated antibody detection.

#### ^*64*^*Cu-BFC-Fab-Trastuzumab*

Moreau et al. (2017) employed ^64^Cu-BFC-Fab-trastuzumab for molecular imaging and biodistribution analyses of mice bearing breast tumor xenografts, achieving a tumor uptake at 24 h after injection ranging from 8.9 to 12.8% ID/g. Using this probe, the most effective visualization was achieved at 4 and 24 h after injection.

#### ^*89*^*Zr∙Df-Fab-PAS200 and *^*124*^*I-Fab-PAS200*

Mendler et al. recently demonstrated the ability of a 200-amino-acid chain composed of Pro, Ala, and Ser residues (PAS200) to effectively bind to Fab antibody fragments while modulating their pharmacokinetic properties so as to enhance that imaging utility by overcoming limitations associated with rapid Fab clearance (Mendler et al. 2015). Using the ^89^Zr∙Df-Fab-PAS200 and ^124^I-Fab-PAS200 probes, these authors were able to detect the tumor-specific uptake of these molecules at 24 h post-injection. Of these two probes, ^124^I was associated with greater thyroid uptake while greater renal ^89^Zr uptake was observed, with ^89^Zr∙Df-Fab-PAS200 exhibiting superior stability and tumor uptake (Mendler et al. 2015).

#### ^*68*^*Ga-NOTA-F(ab')*_*2*_*-Trastuzumab and*^*64*^*Cu-NOTA-Pertuzumab F(ab')*_*2*_

Rathore et al. (2022) performed PET/CT imaging with a ^68^Ga-labeled probe prepared from the Fab domains of trastuzumab that exhibited a molecular weight of just 46.3 kDa, roughly one-third that of the full antibody. Immunohistochemistry was used to successfully confirm the identification of breast and lymphatic lesions detected with this ^68^Ga-NOTA-F(ab')_2_-trastuzumab probe. Suman et al. (2023) also demonstrated that ^68^Ga-NOTA-F(ab')_2_-trastuzumab was capable of identifying tumor cells overexpressing HER2. Lam et al. (2017) similarly evaluated changes in the expression of HER2 over the course of trastuzumab treatment using a ^64^Cu-NOTA-pertuzumab F(ab')_2_ probe, thereby delivering a lower dose of whole-body radiation.

### Imaging probes using engineered antibody fragments and their derivatives

#### Single-chain Fv (scFv)

Noncovalent single-chain Fv (scFv) dimers can be generated by introducing a short (5 amino acid) linker between the variable light (*V*_L_) and variable heavy (*V*_H_) chain domains of an antibody while preventing the homodimeric pairing of the *V*_H_ and *V*_L_ chains. The resultant scFv molecules are capable of simultaneously binding to two target antigen molecules (Robinson et al. 2008). Ueda et al. (2015) demonstrated a high degree of ^68^Ga-Df-anti-HER2 scFv accumulation in mice bearing HER2-positive tumor xenografts such that changes in HER2 status over the course of anti-HER2 treatment could be monitored in a noninvasive fashion.

#### Single-domain antibody fragments (sdAbs) /2Rs15d /nanobody

HER2-targeted nanobodies have been designed for use as molecular imaging probes. Nanosomes are comprised of antigen-binding antibody heavy chain fragments, and are the smallest antigen-binding antibody fragments (12–15 kDa) that exhibit optimal PET/CT imaging properties (Chakravarty et al. 2014).

Small molecule (12–15 kDa) single-domain antibody fragments can serve as ideal tumor contrast agents and are particularly well suited to use with ^18^F. As they are relatively small, however, they are more susceptible than whole mAbs to undesirable changes upon chemical modification that may result in normal tissue retention or altered binding to target proteins (Zhou et al. 2021). In an effort to overcome this limitation, Zhou et al. (2021) employed a thiol-maleimide reaction-based approach to the site-specific ligation of 5F7GGC to a tetrazine-bearing agent. Their resultant ^18^F-5F7GGC single-domain antibody fragment construct was still able to bind HER2 with high affinity and strong immunoreactivity while also undergoing more rapid renal clearance.

In a phase I study focused on ^68^Ga-NOTA-2Rs15d that was conducted by Keyaerts et al. (2016), researchers demonstrated that the radiation dose associated with this probe was comparable to that for other PET/CT tracers in routine use. A high degree of imaging contrast was achieved in both primary and metastatic breast cancer. However, additional phase II trials will be necessary to further evaluate the preferential accumulation of this novel tracer in HER2-positive metastases relative to proximal normal tissues. While it exhibited reduced in vitro and in vivo tumor cell retention, the ability of [^18^F]RL-I-2Rs15d to being a HER2 epitope distinct from that recognized by the HER2-targeted therapeutic antibodies trastuzumab and pertuzumab makes it ideally suited for the PEC/CT-based assessment of the HER2 status of patients who have undergone treatment with either of these mAbs (Zhou et al. 2017).

Mice bearing HER2-positive xenograft tumors have been shown to exhibit strong uptake of the [^18^F]-FB-anti-HER2 nanobody, allowing for the high-contrast imaging of tumors overexpressing HER2. This tracer can also be combined with trastuzumab such that it is ideally positioned for use in patients undergoing treatment with this mAb (Xavier et al. 2016).

### Aptamer-based imaging probes

As short 20–90 base pair single-stranded oligonucleotides, aptamers exhibit strong and selective binding activity such that they can bind to targets of interest via the systematic evolution of ligands by exponential enrichment (Stoltenburg et al. 2007). When using an ^18^F-labeled HER2-targeting aptamer to conduct PET/CT imaging, Kim et al. (2019) demonstrated the ability of this molecule to facilitate cell surface HER2 recognition and to enable the preferential binding of HER2-positive breast cancer cells in vitro and in xenograft-bearing mice. In a similar vein, Gijs et al. (2016) were able to utilize ^68^Ga-labeled NOTA oligonucleotides to conduct molecular imaging, leveraging their advantages over antibodies or traditional proteins including their reduced immunogenicity, smaller size, and less substantial toxicity. These oligonucleotides can be prepared in an inexpensive manner via chemical synthesis and can be readily subjected to chemical modification to enhance their bioavailability, stability, and pharmacokinetic properties (Syed and Pervaiz 2010).

### Engineered scaffold protein-based imaging probes

While antibodies naturally exhibit a high degree of affinity for a wide range of molecular targets, novel affinity ligands can also be developed by leveraging a wider repertoire of natural binding proteins. These novel binding proteins are characterized by a robust scaffold skeleton, ensuring that variable amino acids of interest are stably positioned while minimizing the entropy penalty. Large combinatorial libraries generated via the randomization of unstable amino acids provide an opportunity to select for proteins with specific high-affinity binding activity for targets of interest. These engineered scaffold proteins have been used to develop a range of imaging probes including Affibody molecules, Albumin-binding domain (ABD)-Derived Affinity ProTeins (ADAPT), and Designed ankyrin repeat proteins (Tolmachev and Orlova 2020).

#### Affibody molecules

Affibody molecules are engineered scaffold proteins that are just 58 amino acids in length and that exhibit a high level of affinity for various target proteins. Radiotracer-labeled Affibodies have demonstrated excellent utility in preclinical research when used to sensitively and precisely image target tumors on the day of injection (Tolmachev and Orlova 2020).

Z_HER2:342_ and derivatives thereof are second-generation HER2-targeting antibodies with a wide range of uses. The chimeric Affibody-peptide nucleic acid chimera Z_HER2:342-SR-HP1_ was developed by Honarvar et al. (2016) in an effort to overcome the high levels of renal absorption and consequent reductions in tumor contrast evident for unmodified Affibodies, successfully enhancing radiotracer accumulation within tumors. Building on these promising results, the Z_HER2:342_ derivative DOTA^0^-Z_HER2:342-pep2_ (ABY-002) was designed containing a DOTA chelator conjugated to an N-terminal valine residue to facilitate site-specific radiometal labeling while binding to HER2 with a K_D_ of 65 pmol/L (Orlova et al. 2007). Baum et al. (2010) evaluated three patients that underwent PET/CT imaging using ^111^In- or ^68^Ga-labeled ABY-002 with upfront ^18^F-FDG-PET/CT, revealing that ^111^In- or ^68^Ga-labeled ABY-002 was sufficient to enable the localization of otherwise undetectable metastases and the assessment of their HER2 status.

The recombinant Affibody, ABY-025, recognizes a HER2 epitope located in the extracellular domain III region distinct from that recognized by other HER2-targeting therapeutic agents. ABY-025 also exhibits an excellent tumor/background ratio, enabling the detection of tumor metastases (Sorensen et al. 2014).

Trousil et al. (2014) designed [^18^F]GE-226 (Z_HER2:2891_) as a next-generation Affibody-based radiotracer, amenable to large-scale synthesis while exhibiting superior pharmacokinetic characteristics. [^18^F]GE-226 can function as an imaging probe to differentiate among lesions with varying levels of HER2 expression in metastatic breast cancer patients. However, the efficacy of this probe remains uncertain as imaging results have yet to be published.

#### ADAPT molecules

ADAPT molecules employ a novel scaffolding probe based on the stabilization provided by the Streptococcal Protein G ABD domain. These ADAPT probes can enable the high-contrast imaging of HER2-positive lesions in breast patients (Garousi et al. 2015). In mice bearing human tumor xenografts exhibiting varying HER2 expression levels, DOTA-C^59^-DEAVDANS-ADAPT6-GSSC and DOTA-C^61^-(HE)_3_DANS-ADAPT6-GSSC were both reportedly able to differentiate between tumors expressing low and high levels of HER2 (Lindbo et al. 2018). ^68^Ga-(HE)_3_DANS-ADAPT6-GSSC-NODAGA was also able to serve as an imaging probe with excellent contrast when used for the PER/CT-based evaluation of HER2 status (von Witting et al. 2019).

#### Designed ankyrin repeat proteins (DARPins)

DARPins represent a novel binding molecule class capable of recognizing HER2 or other target proteins of interest with a high degree of selectivity (Zahnd et al. 2007). These proteins contain tightly packed 33-amino acid residues repeats that comprise structural units composed of a β-turn and two antiparallel α-helices, with up to 29 consecutive repeats per DARPin (Walker et al. 2000). These ankyrin repeat domains most often contain 4–6 repeating units, yielding proteins with a right-handed solenoid structure, a continuous hydrophobic core, and a large surface domain accessible by solvents. Researchers have established a DARPin library of molecules with fixed domains important for the structural framework of these proteins and six variable positions per repeat corresponding to nonconserved, surface-exposed residues with the potential to interact with targets (Pluckthun 2015).

DARPins exhibit several attractive properties, as in the case of ^89^ZrDFO-G3-DARPin, which is taken up and retained by tumors while rapidly being cleared from normal tissues, thus yielding PET/CT images with high tumor-to-background contrast (Fay et al. 2022).

### Small molecule peptides

Peptides offer multiple attractive properties that make them well-suited to use as molecular probes for PET/CT imaging. For one, unlabeled small-molecule precursors exhibit well-defined chemical structures, a lack of immunogenicity, regulated pharmacokinetic properties, amenability to a range of modifications, and ease of synthesis that most often occurs via solid-phase peptide synthesis. Following radionuclide labeling, peptides exhibit greater tissue permeability and more rapid blood circulation (Fani et al. 2012; Fosgerau and Hoffmann 2015). Radionuclide-labeled peptides can thus reduce the imaging time necessary to evaluate HER2-positive tumors while accurately informing HER2 expression-based treatment efforts.

#### ^*68*^*Ga- DOTA-(Ser)*_*3*_*-LTVSPWY*

^68^Ga-DOTA-(Ser)_3_-LTVSPWY can specifically accumulate within HER2-positive tumors, enabling the PET/CT-based identification of tumors overexpressing this receptor (Biabani et al. 2021).

#### DOTA-PEG2-GSGKCCYSL (P5) and DOTA-PEG2-DTFPYLGWWNPNEYRY (P6)

Researchers evaluated the HER2 binding activity of ^68^Ga-labeled peptides in vitro and in vivo in xenograft model mice. At 2 h post-injection, they found that [^68^Ga]P5 exhibited a significant increase in binding to HER2-positive tumors as measured in percentage injected dose per gram (%ID/g) relative to tumors negative for HER2 expression (0.24 ± 0.04 vs. 0.12 ± 0.06; *P* < 0.05), while [^68^Ga]P6 exhibited similarly enhanced binding at 1 h post-injection (0.98 ± 0.22 vs. 0.51 ± 0.08; *P* < 0.05). These peptides can thus be leveraged to enable the visual detection of HER2-positive breast tumors (Ducharme et al. 2022).

It has been shown that the ligand portion (including trastuzumab, pertuzumab, affinity, and peptides) of the HER2-targeting radioactive molecular probes is responsible for the specific detection of HER2-positive tumor cells by targeting and binding to the extracellular domains IV or II of HER2 (Carter et al. 1992). Theoretically, alterations in either of the extracellular structural domains IV or II of HER2 could result in negative results on PET/CT molecular imaging targeting HER2, despite the presence of the HER2 receptor. Clinically, this is particularly common in patients with advanced metastatic breast cancer following treatment with frontline trastuzumab and pertuzumab therapy. There are several possible explanations for this. 1. The p95HER2 receptor is a truncated form of the full-length p185HER2 receptor, which lacks the trastuzumab binding site and is therefore unable to detect lesions on HER2-targeted molecular imaging. The p95HER2 receptor is present in about 30% of HER2-positive breast cancers and has been found to be a marker of poor prognosis (Molina et al. 2002). 2. As mucin-4 (MUC4) is a surface glycoprotein, it can physically block the binding sites of mAbs, leading to negative visualization (Oshima et al. 2014; Rowson-Hodel et al. 2018). 3. HER2/neu mutations could theoretically alter the structure of the binding site, inducing off-target effects, although this has not been reported. In summary, structural alterations in the binding site or steric hindrance from surface proteins could affect the binding of the tracer to the extracellular domain of HER2, resulting in false-negative results.

## Clinical applications for HER2-targeted PET/CT scanning in advanced breast cancer

### Early detection of small HER2-positive lesions undetectable via traditional PET/CT

Owing to their ability to specifically bind to HER2, HER2-targeted molecular imaging probes can yield superior specificity and sensitivity when detecting tumors overexpressing these receptor molecules, enabling the clinical detection of small HER2-positive lesions not visible via traditional PET/CT imaging. Dijkers et al. (2010) first reported human PET/CT imaging performed in 14 HER2-positive metastatic breast cancer patients using ^89^Zr-Trastuzumab, revealing that this approach was capable of detecting small liver, lung, bone, and brain metastases that were undetectable via FDG-PET scanning. Laforest et al. (2016) also demonstrated the efficacy of high-dose ^89^Zr-Trastuzumab for PET/CT imaging, while Alhuseinalkhudhur et al. (2020) demonstrated that ^68^Ga-ABY-025 was able to function as an effective imaging probe for dynamic scanning and parametric imaging, mitigating the limited ability of conventional PET/CT scans to detect small liver lesions as a consequence of high levels of background probe uptake such that these metastatic lesions were detectable with greater sensitivity.

### Evaluation of HER2 status in lesions that cannot readily be biopsied

Selecting treatment approaches for tumors that cannot be biopsied, such as deep metastases, brain metastases, and lesions, closely associated with major nerves or large blood vessels is challenging. The application of molecular imaging probes in these cases can provide a more reliable means of determining whether patients are likely to benefit from targeted treatment efforts.

Bensch et al. (2018) employed ^89^Zr-Trastuzumab PET/CT imaging as a means of evaluating the HER2 expression status of 7 breast tumor metastases not accessible for biopsy, enabling more reliable patient dosing. Ulaner et al. (2018) similarly conducted the PET/CT imaging of metastatic breast cancer patients with ^89^Zr-Pertuzumab as a molecular probe, enabling the identification of brain metastases that were not detectable via ^18^F-FDG imaging. Relative to normal brain tissue, this probe exhibited 18-fold higher uptake levels in tumor tissues, potentially owing to the disruption of the blood–brain barrier at metastatic sites (Dijkers et al. 2010). Lee et al. (2017) further demonstrated the utility of ^64^Cu-MM-302 nanoparticles as a PET/CT imaging probe and successfully detected the HER2 status of lesions not readily accessible for biopsy.

Controversy remains regarding the use of ^64^Cu-DOTA-Trastuzumab as a probe for the detection of brain metastases. Tamura et al. (2013) and Kurihara et al. (2015) both successfully applied this probe to detect the HER2 status of metastatic lesions in the brain, consistent with its ability to cross the blood–brain barrier. In contrast, Mortimer et al. (2018) failed to effectively detect brain metastases when utilizing ^64^Cu-DOTA-Trastuzumab for imaging, suggesting that the restricted ability of this antibody-based probe to cross physiological barriers warrants consideration when conducting HER2-targeted PET/CT imaging.

### Evaluating the heterogeneity of multiple metastases

In cancer patients with metastatic disease, metastases are often observed in the form of multiple heterogeneous foci. Treatment planning is generally based on the biological characteristics of one or a small number of these metastases, potentially resulting in the application of therapies that cannot effectively target certain lesions. HER2-targeted PET/CT scanning can simultaneously establish the HER2 status of each of these lesions, thus providing more detailed insight into tumor characteristics and supporting the informed adjustment of patient treatment plans.

Inki (Lee et al. 2022) described the case of a breast cancer patient with HER2-positive disease that experienced local recurrence and pulmonary metastasis following postoperative chemotherapy and dual-target therapy. When they applied ^64^Cu-DOTA-Trastuzumab for whole-body PET/CT imaging, this probe was readily taken up by local tumor tissue in the left breast and left axillary lymph nodes but not by the pulmonary metastases. Consistent with this observation, anti-HER2-targeted treatment resulted in the significant reduction of the left breast and left axillary lesions whereas the lung metastases progressed, providing strong support for the heterogeneous nature of recurrent metastatic lesions. Mortimer et al. (2014) similarly demonstrated the ability of ^64^Cu-DOTA-Trastuzumab to highlight significant variability in the uptake of this probe among lesions in a given patient, with foci lacking any apparent uptake further highlighting the potential for intratumoral HER2 heterogeneity.

### Identifying lesions with altered HER2 status

Much as heterogeneity can exist among lesions in a given patient, so too can HER2 status vary between primary and metastatic lesions. HER2-targeted PET/CT imaging thus provides a basis for potential targeted treatment planning in patients with HER2-negative primary disease.

In their analysis of 20 metastatic breast cancer patients with HER2-negative primary disease, Ulaner et al. (2016, 2017) conducted ^89^Zr-Trastuzumab PET/CT imaging and identified 9 putatively HER2-positive lesions of which 3 were confirmed to be HER2-positive on biopsy while the remaining 5 were false-positives. Similarly, Bensch et al. (2018) used the results of ^89^Zr-Trastuzumab PET/CT imaging to modulate treatment planning in 8 enrolled patients (40% of the overall cohort), including 5 that began anti-HER2 treatment and 3 that did not based on these ^89^Zr-Trastuzumab scans. Ulaner et al. (2020) were also able to apply ^89^Zr-Pertuzumab PET/CT to facilitate the successful detection of HER2-positive metastatic lesions in patients with biopsy-confirmed HER2-negative primary breast tumors. They found that 6 of 24 analyzed patients exhibited suspected HER2-positive lesions upon PET/CT imaging, with 3 of these lesions ultimately being confirmed to be HER2-positive, while 2 were HER2-negative and 1 yielded unspecified results. These results highlight the value of assessing the HER2 status of metastatic lesions such that patients with HER2-negative primary tumors have the potential to benefit from targeted therapy if HER2-positive metastatic lesions are detected.

Sorensen et al. (2016) evaluated 16 metastatic breast cancer patients via ^68^Ga-ABY-025 PET/CT imaging. Based on their results, treatment plans were altered for three patients including one individual with HER2-positive primary tumors but bone metastases exhibiting low levels of HER2 expression and two patients with HER2-negative primary tumors that were found to exhibit high HER2 expression following PET/CT scanning. All of these imaging results were ultimately confirmed via pathological biopsy.

### Evaluating the efficacy of anti-HER-2 drugs

Gebhart et al. (2016) published the results of the multicenter ZEPHIR study of 60 HER2-positive metastatic breast cancer patients in which the association between ^89^Zr-trastuzumab uptake and prognosis was prospectively evaluated following T-DM1 treatment. Patients with HER2-positive lesions, as designated based on the results of ^89^Zr-trastuzumab PET/CT imaging, exhibited greater reductions in tumor size and improved progression-free survival following three T-DM1 treatment cycles. These data highlight the importance of molecular imaging as a noninvasive tool that can enable the dynamic evaluation of tumor responses to particular therapeutic agents, thereby enabling the design and application of antibody–drug conjugates on an individualized basis.

## Poor preclinical-to-clinical translation of PET/CT targeting HER2

While there are currently many kinds of PET/CT molecular probes targeting HER2, difficulties remain in their application in large-scale clinical promotion due to the complexity of the labeling method and the high cost involved. Both PET/CT and SPET/CT can provide dynamic, visualized imaging of both local lesions or whole body imaging using a single drug administration. Compared with PET/CT, SPET/CT has the advantages of having a simple drug-labeling method, high yield, and low price, all of which are advantageous for its clinical promotion. To date, several HER2-targeted SPET/CT tracers have reached the stage of clinical application and have the potential for large-scale clinical promotion. Zhao et al. (2021) developed a novel 99mTc-labeled anti-HER2 single-domain antibody (99mTc-NM-02) and investigated its safety, radiation dosimetry, biodistribution, and tumor-targeting potential in 10 patients with HER2-positive breast cancer. The results showed a mean effective dose of 6.56 × 10 − 3 mSv/MBq, together with good safety and imaging characteristics, with tracer uptake visible in both primary tumors and metastases. Altunay et al. (2023) performed SPET/CT imaging with a 99mTc-labeled single-domain antibody (RAD201) in six patients with HER2-positive breast cancer and found that the tracer was able to discriminate HER2 status in advanced breast cancer, regardless of ongoing HER2-targeted antibody treatment. Cai et al. (2020) in an open-label phase I clinical trial (NCT03546478) involving patients with HER2-positive breast cancer demonstrated specific binding (overall specificity, 60%) of the 99mTc-labeled HER2-targeted affinity ABH2 affibody to target molecules without noticeable adverse effects for the patient.

## Conclusions and outlook

HER2 remains an essential biomarker when assessing patients with breast cancer that guides treatment-related decision-making and prognostic evaluation. HER2-targeted therapies can contribute to significant improvements in survival outcomes for patients with HER2-positive disease, and the ability to accurately evaluate patient HER2 status is thus vital to successful treatment planning. As obtaining pathological specimens can be challenging for certain metastases, HER2 expression levels in primary tumors and metastases may be inconsistent with one another, and different metastases can exhibit heterogeneous phenotypes, HER2 status has the potential to be inaccurately judged through conventional means. HER2-targeted PET/CT imaging, in contrast, offers a noninvasive and comprehensive approach that can enable systemic screening for HER2-positive lesions in real time such that the HER2 status of metastases can be carefully assessed.

At present, mAbs molecular probes are most commonly deployed in clinical studies. These probes exhibit a range of limitations including high levels of nonspecific uptake, undesirably high levels of radiation exposure to health organs, and a risk of false-positive results. As such, protein-based probes, including single-domain antibodies and engineered scaffold proteins, may offer greater utility as molecular imaging agents. These probes are not subject to EPR effects such that the risk of false-positive results can be minimized and HER2 heterogeneity can be effectively assessed and monitored over the course of disease progression. While these findings are promising, future prospective clinical studies will be invaluable as a means of fully evaluating the importance of HER2-targeted molecular imaging in the management of breast cancer.

## Data Availability

Not applicable.

## References

[CR1] Alhuseinalkhudhur A, Lubberink M, Lindman H, Tolmachev V, Frejd FY, Feldwisch J, Velikyan I, Sorensen J (2020) Kinetic analysis of HER2-binding ABY-025 Affibody molecule using dynamic PET in patients with metastatic breast cancer. EJNMMI Res 10(1):21. 10.1186/s13550-020-0603-9 32201920 10.1186/s13550-020-0603-9 PMC7085990

[CR2] Altunay B, Goedicke A, Winz OH, Hertel F, von Mallek D, Meszaros LK, Chand G, Biersack HJ, Stickeler E, Krauss K, Mottaghy FM (2023) 99mTc-labeled single-domain antibody for SPECT/CT assessment of HER2 expression in diverse cancer types. Eur J Nucl Med Mol Imaging 50(4):1005–1013. 10.1007/s00259-022-06066-3 36482076 10.1007/s00259-022-06066-3 PMC9931776

[CR3] Baum RP, Prasad V, Muller D, Schuchardt C, Orlova A, Wennborg A, Tolmachev V, Feldwisch J (2010) Molecular imaging of HER2-expressing malignant tumors in breast cancer patients using synthetic 111In- or 68Ga-labeled affibody molecules. J Nucl Med 51(6):892–897. 10.2967/jnumed.109.073239 20484419 10.2967/jnumed.109.073239

[CR4] Bensch F, Brouwers AH, Lub-de Hooge MN, de Jong JR, van der Vegt B, Schroder CP (2018) ^89^Zr-trastuzumab PET supports clinical decision making in breast cancer patients, when HER2 status cannot be determined by standard work up. Eur J Nucl Med Mol Imaging 45(13):2300–2306. 10.1007/s00259-018-4099-8 30058029 10.1007/s00259-018-4099-8 PMC6208812

[CR5] Biabani AJ, Akhlaghi M, Nikkholgh B, Hosseinimehr SJ (2021) Targeting and imaging of HER2 overexpression tumor with a new peptide-based ^68^Ga-PET radiotracer. Bioorg Chem 106:104474. 10.1016/j.bioorg.2020.104474 33246602 10.1016/j.bioorg.2020.104474

[CR6] Cai J, Li X, Mao F, Wang P, Luo YP, Zheng K, Li F, Zhu ZH (2020) Non-invasive monitoring of HER2 expression in breast cancer patients with ^99m^Tc-Affibody SPECT/CT. Iran J Radiol 17(1):e96419. 10.5812/iranjradiol.96419

[CR7] Carrasquillo JA, Morris PG, Humm JL, Smith-Jones P, Beylergil V, Akurst T, O’donoghue J, Ruan ST, Modi S, Hudis CA, Larson SM (2019) Copper-64 trastuzumab PET imaging: A reproducibility study. Q J Nucl Med Mol Imaging 63(2):191–198 27171605 10.23736/S1824-4785.16.02867-3 PMC9185799

[CR8] Carter P, Presta L, Gorman CM, Ridgway JB, Henner D, Wong WL, Rowland AM, Kotts C, Carver ME, Shepard HM (1992) Humanization of an anti-p185HER2 antibody for human cancer therapy. Proc Natl Acad Sci U S A 89:4285–4289. 10.1073/pnas.89.10.4285 1350088 10.1073/pnas.89.10.4285 PMC49066

[CR9] Chakravarty R, Goel S, Cai WB (2014) Nanobody: The “magic bullet” for molecular imaging? Theranostics 4(4):386–398. 10.7150/thno.8006 24578722 10.7150/thno.8006 PMC3936291

[CR11] Dijkers EC, Oude Munnink TH, Kosterink JG, Brouwers AH, Jager PL, de Jong JR, van Dongen GA, Schroder CP, Lub-de Hooge MN, de Vries EG (2010) Biodistribution of 89Zr-trastuzumab and PET imaging of HER2-positive lesions in patients with metastatic breast cancer. Clin Pharmacol Ther 87(5):586–592. 10.1038/clpt.2010.12 20357763 10.1038/clpt.2010.12

[CR12] Ducharme M, Houson HA, Fernandez SR, Lapi SE (2022) Evaluation of ^68^Ga-radiolabeled peptides for HER2 PET imaging. Diagnostics (basel) 12(11):2710. 10.3390/diagnostics12112710 36359554 10.3390/diagnostics12112710 PMC9689602

[CR13] Fani M, Maecke HR, Okarvi SM (2012) Radiolabeled peptides: Valuable tools for the detection and treatment of cancer. Theranostics 2(5):481–501. 10.7150/thno.4024 22737187 10.7150/thno.4024 PMC3364555

[CR14] Fay R, Toro I, Schinke AL, Simic B, Schaefer JV, Dreier B, Pluckthun A, Holland JP (2022) Sortase-mediated site-specific conjugation and ^89^Zr-radiolabeling of designed ankyrin repeat proteins for PET. Mol Pharm 19(10):3576–3585. 10.1021/acs.molpharmaceut.2c00136 35434995 10.1021/acs.molpharmaceut.2c00136

[CR15] Fosgerau K, Hoffmann T (2015) Peptide therapeutics: current status and future directions. Drug Discov Today 20(1):122–128. 10.1016/j.drudis.2014.10.003 25450771 10.1016/j.drudis.2014.10.003

[CR16] Garousi J, Lindbo S, Nilvebrant J, Astrand M, Buijs J, Sandstrom M, Honarvar H, Orlova A, Tolmachev V, Hober S (2015) ADAPT, a novel scaffold protein-based probe for radionuclide imaging of molecular targets that are expressed in disseminated cancers. Cancer Res 75(20):4364–4371. 10.1158/0008-5472.CAN-14-3497 26297736 10.1158/0008-5472.CAN-14-3497

[CR17] Gebhart G, Lamberts LE, Wimana Z, Garcia C, Emonts P, Ameye L, Stroobants S, Huizing M, Aftimos P, Tol J, Oyen WJG, Vugts DJ, Hoekstra OS, Schroder CP, van Oordt CWM, Guiot T, Brouwers AH, Awada A, de Vries EGE, Flamen P (2016) Molecular imaging as a tool to investigate heterogeneity of advanced HER2-positive breast cancer and to predict patient outcome under trastuzumab emtansine (T-DM1): The ZEPHIR trial. Ann Oncol 27(4):619–624. 10.1093/annonc/mdv577 26598545 10.1093/annonc/mdv577

[CR18] Gijs M, Dammicco S, Warnier C, Aerts A, Impens NREN, D’Huyvetter M, Leonard M, Baatout S, Luxen A (2016) Gallium-68-labelled NOTA-oligonucleotides: An optimized method for their preparation. J Labelled Comp Radiopharm 59(2):63–71. 10.1002/jlcr.3363 26712111 10.1002/jlcr.3363

[CR19] Gonzalez-Angulo AM, Morales-Vasquez F, Hortobagyi GN (2007) Overview of resistance to systemic therapy in patients with breast cancer. Adv Exp Med Biol 608:1–22. 10.1007/978-0-387-74039-3_1 17993229 10.1007/978-0-387-74039-3_1

[CR20] Honarvar H, Westerlund K, Altai M, Sandstrom M, Orlova A, Tolmachev V, Karlstrom AE (2016) Feasibility of affibody molecule-based PNA-mediated radionuclide pretargeting of malignant tumors. Theranostics 6(1):93–103. 10.7150/thno.12766 26722376 10.7150/thno.12766 PMC4679357

[CR21] Keyaerts M, Xavier C, Heemskerk J, Devoogdt N, Everaert H, Ackaert C, Vanhoeij M, Duhoux FP, Gevaert T, Simon P, Schallier D, Fontaine C, Vaneycken I, Vanhove C, De Greve J, Lamote J, Caveliers V, Lahoutte T (2016) Phase I study of 68Ga-HER2-nanobody for PET/CT assessment of HER2 expression in breast carcinoma. J Nucl Med 57(1):27–33. 10.2967/jnumed.115.162024 26449837 10.2967/jnumed.115.162024

[CR22] Kim HJ, Park JY, Lee TS, Song IH, Cho YL, Chae JR, Kang H, Lim JH, Lee JH, Kang WJ (2019) PET imaging of HER2 expression with an 18F-fluoride labeled aptamer. PLoS ONE 14(1):e211047. 10.1371/journal.pone.0211047 10.1371/journal.pone.0211047 PMC6347211 30682091

[CR23] Koleva-Kolarova RG, Oktora MP, Robijn AL, Greuter MJW, Reyners AKL, Buskens E, de Bock GH (2017) Increased life expectancy as a result of non-hormonal targeted therapies for HER2 or hormone receptor positive metastatic breast cancer: a systematic review and meta-analysis. Cancer Treat Rev 55:16–25. 10.1016/j.ctrv.2017.01.001 28288388 10.1016/j.ctrv.2017.01.001

[CR24] Kurihara H, Hamada A, Yoshida M, Shimma S, Hashimoto J, Yonemori K, Tani H, Miyakita Y, Kanayama Y, Wada Y, Kodaira M, Yunokawa M, Yamamoto H, Shimizu C, Takahashi K, Watanabe Y, Fujiwara Y, Tamura K (2015) ^64^Cu-DOTA-trastuzumab PET imaging and HER2 specificity of brain metastases in HER2-positive breast cancer patients. EJNMMI Res 5:8. 10.1186/s13550-015-0082-6 25853014 10.1186/s13550-015-0082-6 PMC4385241

[CR25] Laforest R, Lapi SE, Oyama R, Bose R, Tabchy A, Marquez-Nostra B, Burkemper J, Wright BD, Frye J, Frye S, Siegel BA, Dehdashti F (2016) [^89^Zr]Trastuzumab: Evaluation of radiation dosimetry, safety, and optimal imaging parameters in women with HER2-positive breast cancer. Mol Imaging Biol 18(6):952–959. 10.1007/s11307-016-0951-z 27146421 10.1007/s11307-016-0951-z PMC5096950

[CR26] Lam K, Chan C, Reilly RM (2017) Development and preclinical studies of ^64^Cu-NOTA-pertuzumab F(ab’)_2_ for imaging changes in tumor HER2 expression associated with response to trastuzumab by PET/CT. Mabs 9(1):154–164. 10.1080/19420862.2016.1255389 27813707 10.1080/19420862.2016.1255389 PMC5240646

[CR27] Lee H, Shields AF, Siegel BA, Miller KD, Krop I, Ma CX, LoRusso PM, Munster PN, Campbell K, Gaddy DF, Leonard SC, Geretti E, Blocker SJ, Kirpotin DB, Moyo V, Wickham TJ, Hendriks BS (2017) ^64^Cu-MM-302 positron emission tomography quantifies variability of enhanced permeability and retention of nanoparticles in relation to treatment response in patients with metastatic breast cancer. Clin Cancer Res 23(15):4190–4202. 10.1158/1078-0432.CCR-16-3193 28298546 10.1158/1078-0432.CCR-16-3193 PMC6790129

[CR28] Lee I, Lim I, Byun BH, Kim BI, Choi CW, Lee KC, Kang CM, Seong MK, Kim HA, Noh WC, Lim SM (2022) The prediction of HER2-targeted treatment response using ^64^Cu-tetra-azacyclododecanetetra-acetic acid (DOTA)-trastuzumab PET/CT in metastatic breast cancer: a case report. J Breast Cancer 25(1):69–73. 10.4048/jbc.2022.25.e5 35133094 10.4048/jbc.2022.25.e5 PMC8876541

[CR29] Lindbo S, Garousi J, Mitran B, Vorobyeva A, Oroujeni M, Orlova A, Hober S, Tolmachev V (2018) Optimized molecular design of ADAPT-based HER2-imaging probes labeled with ^111^In and ^68^Ga. Mol Pharm 15(7):2674–2683. 10.1021/acs.molpharmaceut.8b00204 29865791 10.1021/acs.molpharmaceut.8b00204

[CR30] Marquez BV, Ikotun OF, Zheleznyak A, Wright B, Hari-Raj A, Pierce RA, Lapi SE (2014) Evaluation of ^89^Zr-pertuzumab in Breast cancer xenografts. Mol Pharm 11(11):3988–3995. 10.1021/mp500323d 25058168 10.1021/mp500323d PMC4224522

[CR31] Mendler CT, Gehring T, Wester HJ, Schwaiger M, Skerra A (2015) ^89^Zr-labeled versus ^124^I-labeled alphaHER2 Fab with optimized plasma half-life for high-contrast tumor imaging in vivo. J Nucl Med 56(7):1112–1118. 10.2967/jnumed.114.149690 25999431 10.2967/jnumed.114.149690

[CR32] Molina MA, Sáez R, Ramsey EE, Garcia-Barchino MJ, Rojo F, Evans AJ, Albanell J, Keenan EJ, Lluch A, García-Conde J, Baselga J, Clinton GM (2002) NH_2_-terminal truncated HER-2 protein but not full-length receptor is associated with nodal metastasis in human breast cancer. Clin Cancer Res 8(2):347–353 11839648

[CR33] Moreau M, Poty S, Vrigneaud JM, Walker P, Guilemin M, Raguin O, Oudot A, Bernhard C, Goze C, Boschetti F, Collin B, Brunotte F, Denat F (2017) MANOTA: a promising bifunctional chelating agent for copper-64 immunoPET. Dalton Trans 46(42):14659–14668. 10.1039/C7DT01772C 28861553 10.1039/c7dt01772c

[CR34] Mortimer JE, Bading JR, Colcher DM, Conti PS, Frankel PH, Carroll MI, Tong S, Poku E, Miles JK, Shively JE, Raubitschek AA (2014) Functional imaging of human epidermal growth factor receptor 2-positive metastatic breast cancer using ^64^Cu-DOTA-trastuzumab PET. J Nucl Med 55(1):23–29. 10.2967/jnumed.113.122630 24337604 10.2967/jnumed.113.122630 PMC4084518

[CR35] Mortimer JE, Bading JR, Park JM, Frankel PH, Carroll MI, Tran TT, Poku EK, Rockne RC, Raubitschek AA, Shively JE, Colcher DM (2018) Tumor uptake of ^64^Cu-DOTA-trastuzumab in patients with metastatic breast cancer. J Nucl Med 59(1):38–43. 10.2967/jnumed.117.193888 28637802 10.2967/jnumed.117.193888 PMC5750523

[CR36] Nahta R (2012) Molecular mechanisms of trastuzumab-based treatment in HER2-overexpressing breast cancer. ISRN Oncol 2012:428062. 10.5402/2012/428062 23227361 10.5402/2012/428062 PMC3512309

[CR37] Orlova A, Tolmachev V, Pehrson R, Lindborg M, Tran T, Sandstrom M, Nilsson FY, Wennborg A, Abrahmsen L, Feldwisch J (2007) Synthetic affibody molecules: a novel class of affinity ligands for molecular imaging of HER2-expressing malignant tumors. Cancer Res 67:2178–2186. 10.1158/0008-5472.CAN-06-2887 17332348 10.1158/0008-5472.CAN-06-2887

[CR38] Oshima Y, Tanaka H, Murakami H, Ito Y, Furuya T, Kondo E, Kodera Y, Nakanishi H (2014) Lapatinib sensitivities of two novel trastuzumab-resistant HER2 gene-amplified gastric cancer cell lines. Gastric Cancer 17(3):450–462. 10.1007/s10120-013-0290-6 23948998 10.1007/s10120-013-0290-6

[CR39] Pluckthun A (2015) Designed ankyrin repeat proteins (DARPins): Binding proteins for research, diagnostics, and therapy. Annu Rev Pharmacol Toxicol 55:489–511. 10.1146/annurev-pharmtox-010611-134654 25562645 10.1146/annurev-pharmtox-010611-134654

[CR40] Priedigkeit N, Hartmaier RJ, Chen YJ, Vareslija D, Basudan A, Watters RJ, Thomas R, Leone JP, Lucas PC, Bhargava R, Hamilton RL, Chmielecki J, Puhalla SL, Davidson NE, Oesterreich S, Brufsky AM, Young L, Lee AV (2017) Intrinsic subtype switching and acquired ERBB2/HER2 amplifications and mutations in breast cancer brain metastases. JAMA Oncol 3(5):666–671. 10.1001/jamaoncol.2016.5630 27926948 10.1001/jamaoncol.2016.5630 PMC5508875

[CR41] Rathore Y, Shukla J, Laroiya I, Deep A, Lakhanpal T, Kumar R, Singh H, Bal A, Singh G, Thakur KG, Mittal BR (2022) Development 68Ga trastuzumab Fab and bioevaluation by PET imaging in HER2/neu expressing breast cancer patients. Nucl Med Commun 43(4):458–467. 10.1097/MNM.0000000000001521 35131966 10.1097/MNM.0000000000001521

[CR42] Robinson MK, Shaller C, Garmestani K, Plascjak PS, Hodge KM, Yuan QA, Marks JD, Waldmann TA, Brechbiel MW, Adams GP (2008) Effective treatment of established human breast tumor xenografts in immunodeficient mice with a single dose of the alpha-emitting radioisotope astatine-211 conjugated to anti-HER2/*neu* diabodies. Clin Cancer Res 14(3):875–882. 10.1158/1078-0432.CCR-07-1250 18245551 10.1158/1078-0432.CCR-07-1250 PMC2643368

[CR43] Rowson-Hodel AR, Wald JH, Hatakeyama J, O’Neal WK, Stonebraker JR, VanderVorst K, Saldana MJ, Borowsky AD, Sweeney C, Carraway KL 3rd (2018) Membrane Mucin Muc4 promotes blood cell association with tumor cells and mediates efficient metastasis in a mouse model of breast cancer. Oncogene 37(2):197–207. 10.1038/onc.2017.327 28892049 10.1038/onc.2017.327 PMC5930013

[CR44] Sari E, Guler G, Hayran M, Gullu I, Altundag K, Ozisik Y (2011) Comparative study of the immunohistochemical detection of hormone receptor status and HER-2 expression in primary and paired recurrent/metastatic lesions of patients with breast cancer. Med Oncol 28(1):57–63. 10.1007/s12032-010-9418-2 20099049 10.1007/s12032-010-9418-2

[CR45] Scheuer W, Friess T, Burtscher H, Bossenmaier B, Endl J, Hasmann M (2009) Strongly enhanced antitumor activity of trastuzumab and pertuzumab combination treatment on HER2-positive human xenograft tumor models. Cancer Res 69(24):9330–9336. 10.1158/0008-5472.CAN-08-4597 19934333 10.1158/0008-5472.CAN-08-4597

[CR46] Sorensen J, Sandberg D, Sandstrom M, Wennborg A, Feldwisch J, Tolmachev V, Astrom G, Lubberink M, Garske-Roman U, Carlsson J, Lindman H (2014) First-in-human molecular imaging of HER2 expression in breast cancer metastases using the 111In-ABY-025 affibody molecule. J Nucl Med 55(5):730–735. 10.2967/jnumed.113.131243 24665085 10.2967/jnumed.113.131243

[CR47] Sorensen J, Velikyan I, Sandberg D, Wennborg A, Feldwisch J, Tolmachev V, Orlova A, Sandstrom M, Lubberink M, Olofssn H, Carlsson J, Lindman H (2016) Measuring HER2-receptor expression in metastatic breast cancer using [^68^Ga]ABY-025 Affibody PET/CT. Theranostics 6(2):262–271. 10.7150/thno.13502 26877784 10.7150/thno.13502 PMC4729774

[CR48] Stoltenburg R, Reinemann C, Strehlitz B (2007) SELEX–a (r)evolutionary method to generate high-affinity nucleic acid ligands. Biomol Eng 24(4):381–403. 10.1016/j.bioeng.2007.06.001 17627883 10.1016/j.bioeng.2007.06.001

[CR49] Suman SK, Mukherjee A, Pandey U, Chakraborty A, Rakshit S, Tawate M, Dev Sarma H (2023) ^68^Ga-labeled trastuzumab fragments for ImmunoPET imaging of human epidermal growth factor receptor 2 expression in solid cancers. Cancer Biother Radiopharm 38(1):38–50. 10.1089/cbr.2022.0042 36413344 10.1089/cbr.2022.0042

[CR50] Sung H, Ferlay J, Siegel RL, Laversanne M, Soerjomataram I, Jemal A, Bray F (2021) Global cancer statistics 2020: GLOBOCAN estimates of incidence and mortality worldwide for 36 cancers in 185 countries. CA Cancer J Clin 71(3):209–249. 10.3322/caac.21660 33538338 10.3322/caac.21660

[CR51] Syed MA, Pervaiz S (2010) Advances in aptamers. Oligonucleotides 20(5):215–224. 10.1089/oli.2010.0234 20677985 10.1089/oli.2010.0234

[CR52] Tamura K, Kurihara H, Yonemori K, Tsuda H, Suzuki J, Kono Y, Honda N, Kodaira M, Yamamoto H, Yunokawa M, Shimizu C, Hasegawa K, Kanayama Y, Nozaki S, Kinoshita T, Wada Y, Tazawa S, Takahashi K, Watanabe Y, Fujiwara Y (2013) 64Cu-DOTA-trastuzumab PET imaging in patients with HER2-positive breast cancer. J Nucl Med 54(11):1869–1875. 10.2967/jnumed.112.118612 24029656 10.2967/jnumed.112.118612

[CR53] Tolmachev V, Orlova A (2020) Affibody molecules as targeting vectors for PET imaging. Cancers (basel) 12(3):651. 10.3390/cancers12030651 32168760 10.3390/cancers12030651 PMC7139392

[CR54] Trousil S, Hoppmann S, Nguyen QD, Kaliszczak M, Tomasi G, Iveson P, Hiscock D, Aboagye EO (2014) Positron emission tomography imaging with 18F-labeled ZHER2:2891 affibody for detection of HER2 expression and pharmacodynamic response to HER2-modulating therapies. Clin Cancer Res 20(6):1632–1643. 10.1158/1078-0432.CCR-13-2421 24493830 10.1158/1078-0432.CCR-13-2421

[CR55] Ueda M, Hisada H, Temma T, Shimizu Y, Kimura H, Ono M, Nakamoto Y, Togashi K, Saji H (2015) Gallium-68-labeled anti-HER2 single-chain Fv fragment: Development and *in vivo* monitoring of HER2 expression. Mol Imaging Biol 17(1):102–110. 10.1007/s11307-014-0769-5 25049073 10.1007/s11307-014-0769-5

[CR56] Ulaner GA, Hyman DM, Ross DS, Corben A, Chandarlapaty S, Goldfarb S, McArthur H, Erinjeri JP, Solomon SB, Kolb H, Lyashchenko SK, Lewis JS, Carrasquillo JA (2016) Detection of HER2-positive metastases in patients with HER2-negative primary breast cancer using 89Zr-trastuzumab PET/CT. J Nucl Med 57(10):1523–1528. 10.2967/jnumed.115.172031 27151988 10.2967/jnumed.115.172031 PMC5050126

[CR57] Ulaner GA, Hyman DM, Lyashchenko SK, Lewis JS, Carrasquillo JA (2017) 89Zr-trastuzumab PET/CT for detection of human epidermal growth factor receptor 2-positive metastases in patients with human epidermal growth factor receptor 2-negative primary breast cancer. Clin Nucl Med 42(12):912–917. 10.1097/RLU.0000000000001820 28872549 10.1097/RLU.0000000000001820 PMC5708879

[CR58] Ulaner GA, Lyashchenko SK, Riedl C, Ruan ST, Zanzonico PB, Lake D, Jhaveri K, Zeglis B, Lewis JS, O’Donoghue JA (2018) First-in-human human epidermal growth factor receptor 2-targeted imaging using ^89^Zr-oertuzumab PET/CT: Dosimetry and clinical application in patients with breast cancer. J Nucl Med 59(6):900–906. 10.2967/jnumed.117.202010 29146695 10.2967/jnumed.117.202010 PMC6004559

[CR59] Ulaner GA, Carrasquillo JA, Riedl CC, Yeh R, Hatzoglou V, Ross DS, Jhaveri K, Chandarlapaty S, Hyman DM, Zeglis BM, Lyashchenko SK, Lewis JS (2020) Identification of HER2-positive metastases in patients with HER2-negative primary breast cancer by using HER2-targeted ^89^Zr-Pertuzumab PET/CT. Radiology 296(2):370–378. 10.1148/radiol.2020192828 32515679 10.1148/radiol.2020192828 PMC7543717

[CR60] von Witting E, Garousi J, Lindbo S, Vorobyeva A, Altai M, Oroujeni M, Mitran B, Orlova A, Hober S, Tolmachev V (2019) Selection of the optimal macrocyclic chelators for labeling with ^111^In and ^68^Ga improves contrast of HER2 imaging using engineered scaffold protein ADAPT6. Eur J Pharm Biopharm 140:109–120. 10.1016/j.ejpb.2019.05.008 31082509 10.1016/j.ejpb.2019.05.008

[CR61] Walker RG, Willingham AT, Zuker CS (2000) A drosophila mechanosensory transduction channel. Science 287(5461):2229–2234. 10.1126/science.287.5461.2229 10744543 10.1126/science.287.5461.2229

[CR62] Xavier C, Blykers A, Vaneycken I, D’Huyvetter M, Heemskerk J, Lahoutte T, Devoogdt N, Caveliers V (2016) ^18^F-nanobody for PET imaging of HER2 overexpressing tumors. Nucl Med Biol 43(4):247–252. 10.1016/j.nucmedbio.2016.01.002 27067045 10.1016/j.nucmedbio.2016.01.002

[CR63] Zahnd C, Wyler E, Schwenk JM, Steiner D, Lawrence MC, McKern NM, Pecorari F, Ward CW, Joos TO, Pluckthun A (2007) A designed ankyrin repeat protein evolved to picomolar affinity to Her2. J Mol Biol 369(4):1015–1028. 10.1016/j.jmb.2007.03.028 17466328 10.1016/j.jmb.2007.03.028

[CR64] Zhao LZ, Liu CC, Xing Y, He J, O’Doherty J, Huang WH, Zhao JH (2021) Development of a ^99m^Tc-Labeled single-domain antibody for SPECT/CT assessment of HER2 expression in breast cancer. Mol Pharm 18(9):3616–3622. 10.1021/acs.molpharmaceut.1c00569 34328338 10.1021/acs.molpharmaceut.1c00569

[CR65] Zhou ZY, Vaidyanathan G, McDougald D, Kang CM, Balyasnikova I, Devoogdt N, Ta AN, McNaughton BR, Zalutsky MR (2017) Fluorine-18 labeling of the HER2-targeting single-domain antibody 2Rs15d using a residualizing label and preclinical evaluation. Mol Imaging Biol 19(6):867–877. 10.1007/s11307-017-1082-x 28409338 10.1007/s11307-017-1082-x PMC5662479

[CR66] Zhou ZY, Meshaw R, Zalutsky MR, Vaidyanathan G (2021) Site-specific and residualizing linker for ^18^F labeling with enhanced renal clearance: application to an anti-HER2 single-domain antibody fragment. J Nucl Med 62(11):1624–1630. 10.2967/jnumed.120.261446 33637584 10.2967/jnumed.120.261446 PMC8612331

